# Divergence in Corn Mycorrhizal Colonization Patterns Due to Organic Treatment

**DOI:** 10.3390/plants10122760

**Published:** 2021-12-14

**Authors:** Victoria Pop-Moldovan, Rodica Vârban, Larisa Corcoz, Anca Pleșa, Vlad Stoian, Roxana Vidican

**Affiliations:** 1 Department of Microbiology, Faculty of Agriculture, University of Agricultural Sciences and Veterinary Medicine Cluj-Napoca, Calea Mănăştur 3-5, 400372 Cluj-Napoca, Romania; victoria.pop@usamvcluj.ro (V.P.-M.); larisa.corcoz@usamvcluj.ro (L.C.); roxana.vidican@usamvcluj.ro (R.V.); 2 Department of Botany, Faculty of Agriculture, University of Agricultural Sciences and Veterinary Medicine Cluj-Napoca, Calea Mănăştur 3-5, 400372 Cluj-Napoca, Romania; 3 Department of Grasslands and Forage Crops, Faculty of Agriculture, University of Agricultural Sciences and Veterinary Medicine Cluj-Napoca, Calea Mănăştur 3-5, 400372 Cluj-Napoca, Romania; anca.plesa@usamvcluj.ro

**Keywords:** *Zea mays*, arbuscular mycorrhiza, colonization patterns, phenological dynamics, MycoPatt

## Abstract

Excessive application of chemical fertilizers and other agrochemicals can cause large imbalances in soils and agricultural ecosystems. In this context, mycorrhizae represent a viable solution to mitigate these negative effects. Arbuscular mycorrhizae are vital symbionts due to the multiple benefits they bring to both crops and the entire agroecosystem. The main purpose of this study was to observe whether differentiated fertilization has an influence on mycorrhizal colonization patterns in corn. Observed frequencies and intensities of colonization varied widely between phenophases and treatments, with 20% variation for frequency and 14% for intensity, which implies the constant development of both partners during the vegetation period. Arbuscules and vesicles were present in all development stages, but the overall mean was lower than 4% for arbuscules and 1% for vesicles in the analyzed root fragments. Intensity was highly correlated with frequency of colonization compared with arbuscules, where the coefficient was 0.54, and vesicles, with a coefficient of 0.16. Both PCA and NMDS provided good graphical solutions, with a high resolution due to explained variance and good spatial position of vectors. The use of mycorrhizal maps permits the full exploration of colonization patterns and fungal strategy, and the assessment of mycorrhizae-free areas. For the untreated variant, the strategy was oriented toward a longitudinal colonization followed by an irregular development of hyphae with multiple non-colonized areas. Treatment acts to stimulate the appearance of mycorrhizal spots, which further develop radially.

## 1. Introduction

The main domain of food production in the world is agriculture. Over time, agriculture has become fundamental to mankind, as well as to the development of any human activity. It is able to provide food and many resources to the ever-growing world population. Agriculture improves the quality of the environment [1], efficiently disposes of non-renewable resources, provides satisfaction for human needs, and supports economic continuity [2]. However, in recent years, anthropogenic activities have influenced the increase of certain environmental problems [3]. Corn is a globally important crop plant for human consumption [4], but also for animal feed; it depends on a high intake of mineral fertilizers [5]. Due to population growth, but also to the excessive use of agricultural land, it is necessary to apply efficient fertilizer techniques to increase crop productivity [6]. Conventional production is based on the use of chemical fertilizers [7], which can increase crop yields, but can also degrade soils, leading to certain losses at the agroecosystem level [8].

In order to obtain crop productivity, certain factors must be taken into account, one of which is the quality of the soil [9]. Soil quality can influence the entire crop, so the soil must be rich in nutrients and have proper depth and moisture for the plant to grow vigorously [10]. Inadequate fertilization can deplete the soil, resulting in crops with an increased deficit of nutrients. Proper management will lead to a high crop yield. The Common Agriculture Policies try to mitigate the current issue in corn crops, which, in recent years, present an increase both in productivity and the consumption of synthetic fertilizers. One of the most sustainable solutions is to increase the use of biological solutions on a larger scale, especially those related to soil diversity and rhizosphere augmentation. The most important issues related to agriculture are climate changes [11], environmental pollution, and an accelerated degradation of ecosystems and therefore the disappearance of soil biodiversity [12]. The most important limiting factor of crop productivity is the availability of mineral nutrients in the soil. Excessive application of chemical fertilizers can cause major imbalances leading to massive ecological degradation throughout the belowground environment [13]. Therefore, by improving the efficient use of nutrients in crops, we can reduce the level of fertilizers used to achieve productivity [14].

Long term application of mineral fertilizers can cause acidification of soils and also an increase in heavy metals in both soil and plant systems [15]. A better option for the environment is the use of soil microorganisms such as one of the most important symbionts—mycorrhizae [16]—which are able to colonize plant roots and increase soil productivity and, implicitly, obtained yields [17]. Soil biodiversity is essential for the activity of terrestrial ecosystems [18]. Microorganisms present in soils have many roles in both soil substrate and plants. Soil fertility is a benefit of microorganisms that results in increased production capacity [19]. The conservation and improvement of agroecosystems is closely related to the diversity and abundance of soil microbiomes. A decrease in the number of soil microorganisms is visible in the decline of agroecosystem functioning. Crop productivity and food security are also affected by climate change and agronomists need to adapt by better using the ecosystem services, but also the biodiversity the ecosystem offers [20]. This is a practice by which the use of chemical fertilizers can be reduced without changing plant nutrition and, in addition, it can even increase the available nutrients for plants. When microorganisms come into contact with plant roots, they modify the plant’s physiological processes and act as growth and development promoters [21].

The rhizosphere is the living environment for a large community of microorganisms, being the thin substrate of the soil in the immediate vicinity of the roots [22] where most interactions between microorganisms and plant roots are found [23]. Soil microorganisms have important roles in terms of conserving biodiversity, soil fertility, and flora. A special group of microorganisms form symbiotic associations with about 70% of the roots of vascular plants [24,25]. This group of microorganisms is represented by mycorrhizae [26]. Mycorrhizae are widespread around the globe, having been present since 400 million years ago. Most terrestrial plants benefit from the association with mycorrhizal fungi, but some have become dependent on them [27]. Mycorrhizae help plants to grow and develop, thus facilitating the absorption of nutrients, especially phosphorus [28]. The fungus receives the carbon produced by plants as a result of photosynthesis and the plant receives nutrients to which its roots do not have access from the fungus [29].

Fungal hyphae are much thinner and longer than the roots of plants [30], so they can reach hard to reach areas to provide the plant with nutrients [31]. Mycorrhizal fungi explore a large area of soil, so changes occur in soil fertilization. Therefore, they contribute to the absorption, transfer, and permanent movement of nutrients in nature [32]. Modern agriculture is trying to increase the productivity of agricultural crops by reducing the amount of chemical fertilizers, pesticides, and herbicides used. Arbuscular mycorrhizae are essential pawns in organic farming due to the many benefits they bring to host plants, soil, and the entire ecosystem [33].

Our paper aims to assess several segments of mycorrhizal mechanisms in corn roots, which are condensed as: the evaluation of the impact of organic inputs on mycorrhizal colonization and the evaluation of the level of mycorrhizal colonization in maize plants in different growth phenophases. The expected results follow the specific pattern of mycorrhizal colonization under the influence of organic inputs and the specificity of colonization in different phenophases. 

In order to achieve the proposed objectives and to understand mycorrhizal colonization potential, we propose two hypotheses: (i) mycorrhizal colonization shows differences induced by fertilization or by the phenophase of plant development, and a divergence of colonization during the vegetation period; (ii) mycorrhizae show maximum colonization in a specific phenophase and have different colonization strategies during the vegetation period.

## 2. Results

### 2.1. Assessment of Normality and Distribution of Colonization Data

The entire data analysis starts with a pairwise comparison of data distribution based on histograms, developed for each of the first experimental factors: control vs. organic treatment (Figure S1a–m). This analytical process acts as the first level in the establishment of colonization strategy, which is further divided by the second factor (plant phenophase). Colonization frequency presents a similar pattern of data distribution (Figure S1a,b), with more than 800 observations in the range 0–10%. However, a more detailed analysis shows an increase of 100 observations for the treated variant compared to the control in the frequency range 10–20%. These supplementary observations are equally divided in the range 40–100% in the case of the control variant. The intensity of colonization presents a very different image of mycorrhizal extension in roots (Figure S1c,d) between the two treatments. For the control variant, the maximum intensity observed in root fragments was 80%, compared to the treated variant, in which there were areas with almost 100% intensity. Furthermore, only 800 observations were in the range 0–10% in the control case, compared to almost 1500 in the case of the treated variant. For the next range (10–20%), both variants presented a similar number of observations. Mycorrhizal colonization lacked arbuscule development, with these structures being less than 5% of the total colonization in almost 90% of our observations (Figure S1e,f), regardless of the applied treatment. An interesting case is that a very limited number of observations showed more than 50% of the mycorrhized area with arbuscules. Vesicles followed a trend similar to arbuscules, with more than 90% of the observations being in the range 0–2% of presence. These structures occupied 13% of mycorrhized area at maximum development, in the case of the control, and just 9% in the case of the treated variant. The non-mycorrhized areas showed distinct distribution patterns (Figure S1i,j), with more than 2/3 of the observations of the treated variant in the range 90–100%. In contrast, the same parameter in the control variant presents a more balanced colonization, sustained by the presence of complete non-colonized areas in only half of the observations. The most different histogram of observations is the one that presents the ratio between mycorrhizal and non-mycorrhiza areas (Figure S1m,n). This report shows the maximum share of the roots that is allocated to mycorrhizal development. For the control variant, the maximum value was 3.5, with more than 2000 observations in the range 0–0.5. For the treated variant, almost all observations were in the range 0–2.5, but some of the observations show over 5 and up to 35. In these cases, entire root fragments were colonized by hyphae, with or without development of arbuscules or vesicles.

### 2.2. Variance Analysis and Post-Hoc Exploration of Results Related to Mycorrhizal Colonization

The next step in order to obtain the results is the analysis of the variance. The analysis of the treatment–phenophase interaction indicates a very significant impact at the level of each factor, but with a reduction of impact in the case of synergistic action (Table 1). In the case of colonization frequency, the variations were extremely large between phenophases and between treatments. The maximum of this parameter was 50%, reached in the non-fertilized variant at the end of the vegetation period. In the case of this variant, the increases in colonization frequency were progressive, with a maximum of 10% between B3 and B4. In the case of fertilization, the initial increase was 10 percent (B1–B2), followed by 15 percent (B2–B3), but with a reduction in Phenophase B4. Overall, in this case the final frequency of colonization was 13% lower than in the case of the unfertilized control. For intensity, there was an increase simultaneous with the development of the plants, the maximum being in the case of the fertilized variant (A1–B5, 20.25%). The differences were significant between fertilized and unfertilized, with a light but significant weight in favor of fertilization for Phenophases B2 and B3. On the other hand, there was a change in favor of mycorrhization in non-fertilized variants in Phenophases B4 and B5. The difference of 4–5% between fertilized and unfertilized is sufficient to ensure plant nutrition. In the case of arbuscules, the highest value was registered in the unfertilized variant, 3.67%, in the phenophase of cob formation. The vesicles had approximately the same value of 0.11% in the case of fertilized variants with 6 leaves and 8–10 leaves. The maximum of 0.28% was measured in variant A1 B3. This phenomenon is due to poor storage of reserves in the vesicles. The degree of colonization follows a slight increase from the moment A0, registered in the non-fertilized variant. At physiological maturity of the plant, the highest value was 13.3%. The non-mycorrhizal areas are found predominantly in the first phenophases of the plant and in the non-fertilized variants, with percentages of 93.9% for A0–B1 and 90.9% for A1–B2.

### 2.3. Correlation of Mycorrhizal Colonization Parameters

The differences between the colonization parameters support both the direct impact on the colonization of the treatments applied and the indirect impact of the structure production strategy. The use of correlations between colonization parameters is useful to describe the biological mechanism of colonization in a complex way (Table 2). All correlations are significant, which shows the close link between the colonization strategy and the developed structures. The maximum correlation is between frequency and intensity (0.91), which indicates intra-root development in almost all cases of root penetration. The observed extension is very strongly connected with the development of arbuscules. However, the vesicles show a lower weight of correlation, being little correlated with frequency, but having a similar weight of correlation between the arbuscules and the intensity of the colonization. The degree of colonization is similarly correlated with frequency and arbuscules, but the maximum dependence is on the intensity of colonization. Minimum correlation occurs between vesicles and frequency (0.16), which indicates a strategy of colonization of an annual root system involving reduced storage of nutrient reserves. 

### 2.4. Analysis of the Frequency–Intensity Interaction of Colonization and the Prognosis of the Development of Mycorrhizal Systems in the Root Cortex

**Figure 1:**
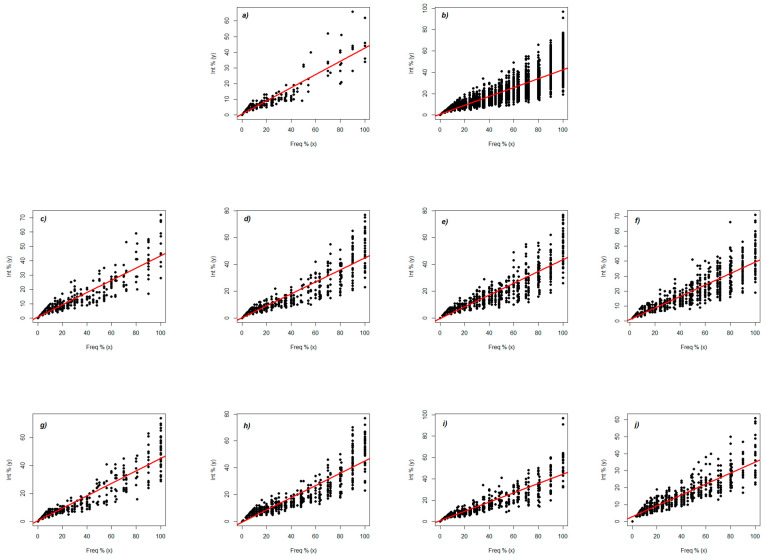
Analysis of the frequency–intensity interaction of colonization and the forecast of mycorrhizal system development in root cortex separated by phenophase and applied inputs: (**a**) A0–B1; (**b**) all samples; (**c**) A1–B2; (**d**) A1–B3; (**e**) A1–B4; (**f**) A1–B5; (**g**) A2–B2; (**h**) A2–B3; (**i**) A2–B4; (**j**) A2–B5. A0–B1 represents the phenophase of 2–4 leaves (as a control point for the start of mycorrhizal colonization). The application of fertilizer leads to four different combinations for each variant (A1—control (unfertilized variant) and A2—treated variant): B2—6 leaves; B3—8–10 leaves; B4—cob formation; B5—physiological maturity. Freq—frequency of colonization (%), Int—intensity of colonization (%).

The analysis of the frequency–intensity interaction of the colonization and the prognosis of the development of the mycorrhizal system in the root cortex was performed with the help of scatter plot graphs (Figure 1a–j, Table 3). The evaluation of the intensity–frequency relationship of colonization indicates a strong variation both at the level of the grouping of experimental factors and relative to the positioning and advance in the roots. For A0–B1, frequencies in the range 0–40% are associated with intensities in the range 0–20%, following the normal development curve (Figure 1a). Above these levels, there is a discontinuous colonization, which lacks linearity. The regression equation sets a base level of intensity of 0.46, to which is added a value of 0.42 for every 1% of the frequency. The value of 0.42 is maintained for the whole experiment and associated with 1% of the frequency (Figure 1b), but the basic value changes to 0.59. For A1–B2, the regression equation sets a basic intensity level of 0.67, to which is added a value of 0.43 (Figure 1c). In the range of 0–40%, the frequency of colonization is tight, followed by the extension of the results in the intensity range of 30–60%. In variant A1–B3, the results are similar, but if we look at the regression equation, it establishes a basic level of intensity of 0.20, to which is added the value of 0.45 (Figure 1d). The regression equation of variant A1 B4 sets a basic level of intensity of −0.24, to which is added the value of 0.44 (Figure 1e). For variant A1–B5, the highest basic value (1.11) is found in the regression equation, to which is added 0.38, giving the highest value in the non-fertilized variant (Figure 1f).

The observations are very close to the normal line, in the range of 0–40%. The stability and cohesion of colonization extends to a frequency value of 70%. In variants A2–B2 and A2–B3, a group of observations was observed in the first range 0–20% (Figure 1g,h, Table 3); in both variants, they are very close to the transverse line. The regression equation establishes a level of intensity of 0.46 for Variant A2 B2, to which is added 0.45; for Variant A2 B3, the level of intensity is 0.56, to which is added 0.46. Variant A2 B4 presents a regression equation in which a level of intensity of 0.82 is established, to which the value of (0.44) is added, which shows us that the results are mostly grouped in the first ranges, namely 0–20% (Figure 1i). For variant A2–B5, the regression equation has the highest base value for the fertilized variant, a level of 2.78, to which is added the value of 0.32% for every 1% of the frequency (Figure 1j). Most of the results are at the level of the normal line; a dispersion then appears.

### 2.5. Spatial Exploratory Analysis of Colonization Based on PCA vs. NMDS Ordinations

**Figure 2:**
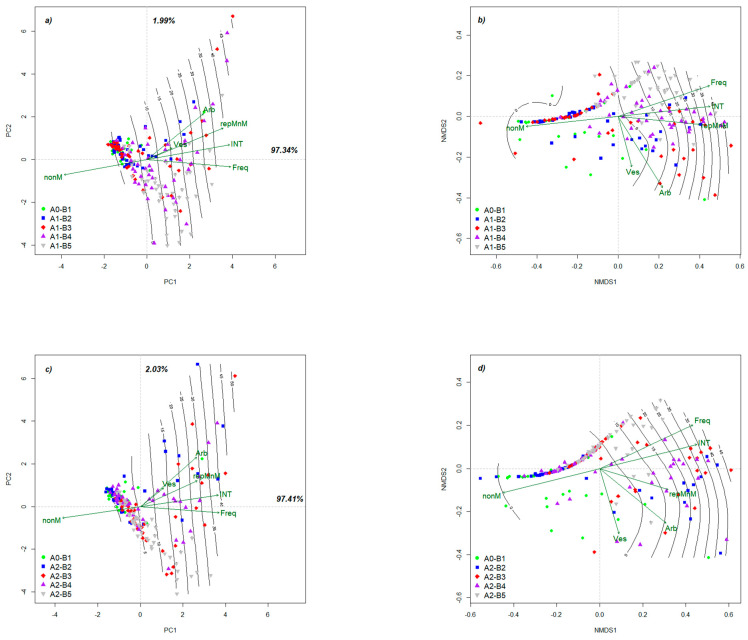
Spatial exploratory analysis of colonization based on PCA vs. NMDS ordinations: (**a**) PCA of A0–A1; (**b**) NMDS of A0–A1; (**c**) PCA of A0–A2; (**d**) NMDS of A0–A2. Legend: A0–A1—data derived from the unfertilized variant + data from 2–4-leaf phenophase; A0–A2—data derived from the fertilized variant + data from 2–4-leaf phenophase. A0–B1 represents the phenophase of 2–4 leaves (as a control point for the start of mycorrhizal colonization). The application of fertilizer leads to four different combinations for each variant (A1—control (unfertilized variant) and A2—treated variant): B2—6 leaves; B3—8–10 leaves; B4—cob formation; B5—physiological maturity. Freq—frequency of colonization, INT –intensity of colonization, Arb—arbuscules, Ves—vesicles, nonM—non-mycorrhizal areas, repMnM—mycorrhizal/non-mycorrhizal report. Isolines plotted on the ordination represent data of colonization degree recorded for each variant. Variance explained in PCA is written at the end of each axis.

The PCA analysis allows both the exploration of observations on mycorrhizal colonization and the projection of colonization parameters relative to the synthetic index: the degree of colonization. In addition, the dispersion of the values recorded for each of the factors studied and the spatial projection of the vectors relative to each parameter of the colonization are analyzed (Figure 2a,c). The quality of microscopic observations is supported by antagonistic gradients of intensity and non-colonized areas, with a spatial orientation in the ++ and − quadrants, respectively. The angle formed by the arbuscules and the frequency represent a maximum of 15% of the analyzed data. In other words, in this area of ordination, the change of parameters is very dynamic and involves the whole accumulation of structures developed by mycorrhizae. Most of the recorded data are positioned in the range of 0–10% of the degree of colonization. A second phenomenon observed at the level of ordination is the grouping of these data before the central point, which implies the stability of the colonization phenomenon. From the perspective of the applied treatments, stability is at its maximum in the two-leaf phenophase and in non-fertilization conditions. Simultaneous with the evolution of the mycorrhizal phenomenon, a strong dispersion of data in the ordination plan is visible. From a biological point of view, this indicates differentiated permissiveness at the root level, the potentially colonized areas alternating with the non-colonized areas. In the context of intensity differentiation, the fluctuating value of this parameter is explained independent of the frequency of the colonization phenomenon.

The NMDS type analysis (Figure 2b,d) is similar to the PCA type analysis, but this type of ordination is useful for much easier observation of mycorrhizal colonization at the root level, and also for the specific parameters of the colonization. Both for the control variant and for those variants to which fertilization was applied, the results are relative to the range 0–5% of the degree of colonization, grouped in the left quadrant of the ordination. The frequency and intensity maintain their associated vectors above Axis 1 of the ordination, which indicates the change of these parameters in association. At the opposite pole, the vesicles and arbuscules are associated with the second axis of the ordination, again indicating a change and a presence in the same mycorrhizal areas, but with a much lower presence in the colonization mechanism. A similar alignment to Axis 1 of the vectors intensity and the ratio of mycorrhizae to non-mycorrhizae indicates the dependence of the two parameters and biologically abundant colonization as soon as the fungi have managed to penetrate the root. The phenomenon is important from two perspectives. The first concerns the specific permissiveness of the plants towards root hyphal penetration, which imposes the selection of hybrids with a high degree of association with mycorrhizae and good adaptation to soil conditions. The second perspective is related to the selection of mycorrhizal isolates with strong root penetration capacity and the association with as many hybrids as possible on the market. Unlike PCA, at the NMDS level, the folding of observations on the non-mycorrhizal gradient can be observed, which indicates the existence of completely uncolonized root areas of fungi. Regarding the graph of Variant A0–A2, the ordination of the results differs from the previous variant. Most observations are located in the left quadrant of the order. At the level of this variant, we observe an increase of intensity simultaneously with the frequency of mycorrhizal colonization, but at the same time, an increase of non-mycorrhizal areas, especially in the first stages of plant development. The fungal structures present in the root follow a different course, unlike the previously presented results, which indicates that the plant has accepted the symbiont. The areas where the mycorrhizae are present mostly appear in the phenophases of 6 leaves and 8–10 leaves.

### 2.6. Mycorrhizal Patterns in Corn Roots, Due to the Interaction of Treatments and Phenophase

**Figure 3:**
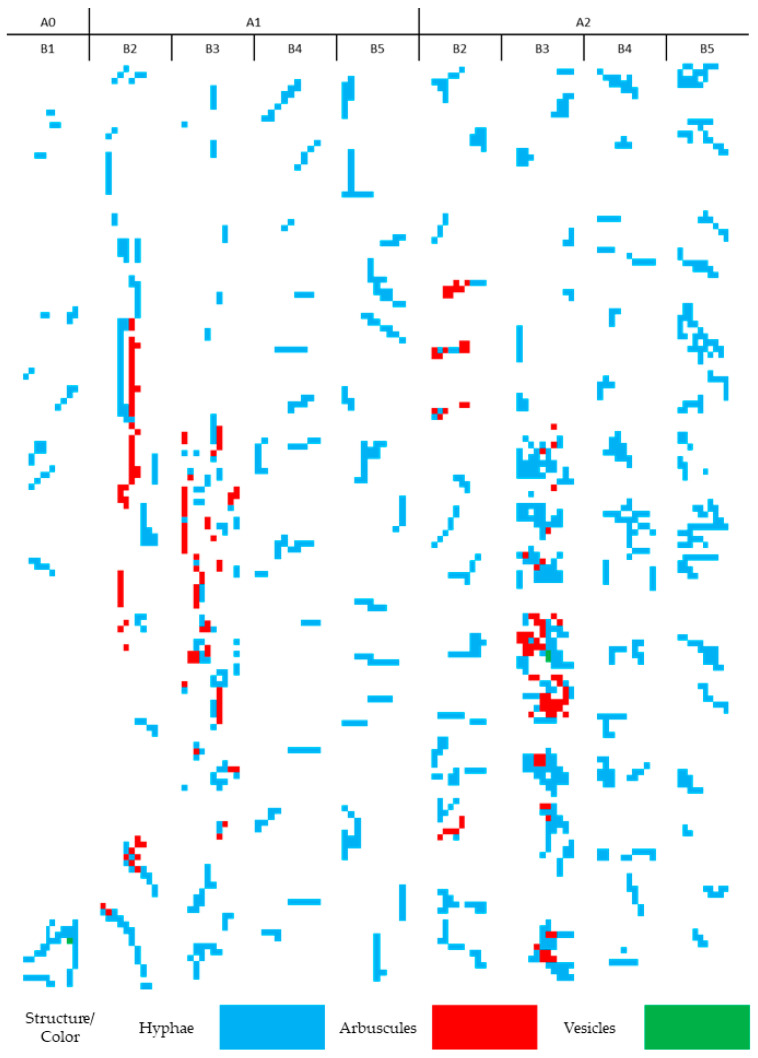
Mycorrhizal patterns in corn roots due to the interaction of treatments and phenophase. A0–B1 represents the phenophase of 2–4 leaves (as a control point for the start of mycorrhizal colonization). The application of fertilizer leads to four different combinations for each variant (A1—control (unfertilized variant) and A2—treated variant): B2—6 leaves; B3—8–10 leaves; B4—cob formation; B5—physiological maturity.

The mycorrhizal pattern is represented by the mycorrhizal map (Figure 3). This representation makes it possible to relatively easily observe the fungal structures present in the corn root. The experiment resulted in a large database, which was merged into 390 maps of mycorrhizal patterns induced by corn treatments in different vegetation phenophases. The methodology for selecting the general patterns associated with each treatment and phenophase of the culture involved strong filtering of the database. Each stage is associated with a colonization parameter related to the general value of the parameter for each experimental variant (Table 1). Thus, based on the averages obtained for the frequency of colonization, only those segments were selected that ranked in a range of 0–5% deviation from the average. Once this point was reached, those maps with intensities in the same range of 0–5% compared to the average were further selected from the filtered segments; this step was followed by the application of the same principle at the level of arbuscules and vesicles. In total, following the four-step filtering procedure, maps showing the general mathematical average pattern of phenophase-induced colonization and treatments could be selected.

The conversion of microscopic observations into colonization maps permits a very precise analysis of colonization patterns associated with both treatments and phenophases (Figure 3). The first colonization pattern, in the 2–4-leaf stage (A0–B1), presented an incipient colonization, with large areas free of fungal structures. Hyphae had reduced development potential and the branching was almost missing. After this point, based on the application of the organic treatment, the analysis of colonization patterns permits the comparison of the two types of symbiosis evolution. In the stage of 6 leaves (B2), the control variant (A1–B2) showed longitudinal development of hyphae, with lateral branching for the formation of arbuscules. The free mycorrhizal areas were present at a lower percentage, and the entire root presents only few areas of hyphal discontinuity. On the other hand, mycorrhizal patterns of the treated variant (A2–B2) showed greater discontinuity of colonization, with colonized spots alternating with mycorrhizae-free areas. Arbuscularity is a localized phenomenon, which represents a strategy for punctual intracellular colonization, instead of intercellular colonization, and the reconnection of hyphae in a network. The next phenophase, 8–10 leaves (B3), showed great divergence between the two variants. In the roots of the control variant (A1–B3), mycorrhizae had longitudinal development, with lateral connections between hyphae and arbuscules developing. Even if the mycorrhizae-free areas are present, the growth direction of the hyphae indicates reconnection developing. For the treated variant (A2–B3), the entire image of colonization was an amplified development of the previous stage. Mycorrhizal spots evolved rather radially, with the development of arbuscules inside the colonized area. There were many discontinuities present between the colonized areas. For the last two phenophases, there was a decrease in colonization, regardless of the applied treatment. The lack of fertilizer in the control variant (A1–B4) drastically reduced colonization; the non-colonized areas occupied a large part of the roots. There was mitigation of this phenomenon in the next phenophase (A1–B5), but the development of hyphae was irregular, and no future connectivity was visible. The application of the treatment maintained the colonization pattern in the roots (A2–B4 and A2–B5) at a lower value compared to previous phenophases, but with a stable development pattern.

## 3. Discussion

The data analysis performed on our results shows different changes in mycorrhizal development in roots and a large variation in colonization parameters due to both phenophase and applied treatments. Furthermore, the entire research permitted different assumptions about the colonization mechanism and the reaction of plant to the development of a fungal partner. All the processes of data analysis were constructed to increase, step by step, the visibility and the understanding of the entire colonization mechanism. Based on histograms of colonization frequency, we observed that 1/3 of the total root fragments presented an incipient colonization (less than 10%) and half of the root fragments had less than a 30% presence of mycorrhizal structures. This mechanism is related to the annual character of roots, which implies continuous growth and areas with differing permissiveness for symbionts. The phenomenon can be explained by the presence of nutrients directly accessible to the plant for a short period of time, which reduced permissiveness towards colonizing fungi, followed by the maintenance of this reduced permissiveness. Colonization intensity shows that mycorrhizal development was more balanced in the control variant compared to the treated variant, where colonized areas of roots were present as spots that were not always further developed. This aspect is due to the appearance of an area of depletion of native resources around the root in the non-fertilized version, which corresponds to a strong investment in plant symbiosis. A possible hypothesis in this case is the reduced capacity of the plants to produce enough new roots, constantly providing optimal space for colonization. The lower presence of both arbuscules and vesicles makes a deeper analysis of their location patterns important. A follow-up study based on these structures can provide important information regarding the mechanisms that block or restrict their proliferation. It is important to correctly identify the restrictor for hyphal penetration into the root cells followed by the development of arbuscules, in order to unlock this potential and to improve the transfer rate of nutrients to plants. Similarly, for vesicles, an increase in their presence is very useful for plant resistance to stress, due to their storage potential. An interesting aspect is the presence and the position of non-mycorrhized areas. Two hypotheses deserve to be taken into account for their presence in order to mitigate this phenomenon. The first one is that roots grow faster than mycorrhizal can develop, which should make these areas less present in the senescent roots analyzed in advanced phenophases. The second hypothesis is related to root permissiveness, which may fluctuate along the entire length; in this case, the colonization is restricted to its first location and fungal structures are not present. Further investigation of this hypothesis requires root analysis during the entire vegetation period and the integration of arbuscules and vesicles in the mycorrhized areas, as well as the identification of the period when colonization is suppressed by the plant. The values registered in the mycorrhizal/non-mycorrhizal report indicate that the roots of the control variant have a permissiveness of under 80% for the space allocated to be colonized by fungal symbionts. This value establishes the native colonization potential of a plant in non-fertilized conditions. When the value increases, it indicates a proliferative phase in the fungal life cycle, which can be associated with many potential cases such s a need for increased transfer of nutrients, the production of a new root set or a branching followed by a secondary root formation phenomenon, an imbalance in plant development due to different types of stress, or the migration of a symbiont toward parasitic nutrition. 

The correlation observed between mycorrhizal parameters shows the annual character of corn, which corresponds to the development of a growing root system and alternating root permissiveness depending on the momentary availability of nutrients. A large number of non-colonized areas are inserted in the intraradicular hyphae network and these have a large influence on the ratio between the areas associated with the symbiont and the areas without hyphae. Another aspect is the permissiveness of type of roots for symbionts, with the colonization capacity being directed towards intraradicular extension. The next step, based on regression analyses, indicates the relevance of sampling during the two-leaf phenophase and the use of these samples as a control for the whole experiment. Separately, for each factor and each phenophase, changes in the regression equations were observed due to the distinct association of intensity with colonization frequency. In the range 0–40%, the results are grouped near the curve, but as the resulting ranges increase, they disperse more and more compared to the transverse line. This shows a stability of colonization, but with a pulsar-type intraradicular development. This phenomenon involves the alternation of newly penetrated areas of the root with those in which intraradicular development is continuous.

Globally, corn is considered a plant with a high value, both industrially, as food, and as feed. Due to corn’s very branched root system, but also to the character of the species, various studies are presented in the following lines that focus on corn colonized by mycorrhizal fungi and the influence of this fungi on host plants in terms of fertilization [34]. A study on mycorrhizal colonization of Bt corn under different applied fertilization conditions observed that fertilizers inhibit the development of mycorrhizae and therefore the colonization mechanism. For this type of corn, the level of colonization and the inoculation potential are strictly related to the applied fertilization system, and symbiosis remains inactive.

Zhao et al. [35], observed that soil fertility, in terms of nitrogen and phosphorus availability, significantly influences the formation of mycorrhizal symbiosis. Furthermore, the richness of *Acaulospora* and *Gigaspora* species increased and that of *Glomus* sp. decreased with the application of P, while the addition of N did not change the composition of fungal mycorrhizal structures. NPK application can have a negative effect on mycorrhizal colonization [36] and species diversity decreased following the application of inputs in shallow soil. The study [37] states that arbuscular mycorrhizae especially sporulate at depths of 50–70 cm and the diversity and richness of species decreases with soil depth.In general, fertilizers have a negative effect on the colonization of mycorrhizae, following a long-term study of differentiated fertilization, both organic and mineral, has produced a negative effect on mycorrhizal fungal communities. Shannon diversity of arbuscular mycorrhizae decreased due to NPK fertilization over this period. The richness of mycorrhizal species was also greater in the unfertilized soil.

Astiko et al. [38] observed that inoculation of maize plants with mycorrhizal fungi and application of N P-based treatments produce an effect on maize cultivation. It was found that P fertilization did not affect the colonization of the corn root. The degree of root colonization was higher within the root to which the NPK mineral fertilizers were applied. The length of the mycelium, but also the number of spores, were significantly higher in corn fertilized with organic fertilizers, and this indicates that organic fertilization is more efficient for production compared to conventional fertilization. Following two experiments, the contribution of native mycorrhizal fungi to maize growth in different states of mineral and organic fertilization in soils with high and low P content was investigated. Mycorrhizal colonization was the weakest in soil where no treatment was applied in the first experiment. The biomass of maize plants was lower in the non-fertilized treatment compared to the other fertilizer treatments. For the second experiment, mycorrhizal colonization was significantly higher in maize plants that were not treated with NK fertilizer. In the experiment with a high P content, both mineral and organic fertilization attenuated the cessation of plant growth caused by arbuscular mycorrhizae. In the soil with low P content, mycorrhizae induced the growth of vegetative plants both without fertilization and with organic and mineral fertilization [8].

Mycorrhizal communities produce various plant benefits; these led to a positive effect on plant biomass, but also on P content of maize. A reduction in colonization was observed in plants to which P fertilizers were applied, showing that colonized plants can acquire essential nutrients and thus save P-based fertilizer, especially in fields where it has been previously applied [39]. Low concentrations of fertilizers with N and P do not significantly affect the development of colonization, but an increase in nutrient concentration acts as a restrictor for this mechanism. Therefore, it can be considered that the application of nutrients in low concentrations does not affect the growth rate of hyphae and the formation of arbuscules and vesicles, nor their long-term maintenance [40].

In a study that followed on effects of N-P fertilization over 27 years to see if there were species that tolerate fertilizers, it was observed that, in general, mycorrhizal sporulation was reduced by fertilization. The reduction was caused due to the accumulation in the soil over time. Thus, the increase of the fertilizer rate from 0–0 to 180–180 kg N-P205 ha/year produced a decrease in the number of spores by 70% [41,42].Martinez and Johnson pointed out that in cloned corn, spores of arbuscular mycorrhizae were much more abundant in plots of land where there was intensive input management as opposed to the adjacent native vegetation [42].

According to Smith and Read [43] and Verbruggen et al. [44], it was found that mycorrhizal colonization as well as the number of spores can be stimulated by changing the agricultural practices and fertilization regime applied, because abundance of species is negatively correlated with intensive agricultural production. Some authors [45,46] consider corn to be a plant that depends on mycorrhizal colonization, especially in soils with low phosphorus content. 

The analysis of colonization patterns is vital for understanding mycorrhizal physiological mechanisms, both in terms of extracting colonization maps and to realistically observe the level and the position of fungal structures in roots. Thus, evaluations can be made regarding the frequency and intensity of structures synthetized in root colonization. The present paper provides observations on changes in colonization patterns due to the effects of organic treatments on maize crop. Patterns of maize colonization can be effective for assessing how plants connect to mycorrhizal nutrient networks in soil, which is very important for plant breeding studies. Through these patterns, the optimal level of native mycorrhizal extension can be established and further developed as an indicator for input applications in agronomy. This realistic information about the mycorrhizal mechanism provides a database that may be necessary for the further development of the Common Agricultural Policy and for the concept of adaptive strategy. The scientific context sustains the development of MycoPatt-like studies due to the digital information provided and the comprehensive analysis of changes in mycorrhizal patterns due to treatments.

## 4. Conclusions

Frequency of colonization varied largely between 13.34% in the 2–4-leaf phenophase, to a value of 50.17% for the control variant and 37.33% for the treated variant, both at the end of the vegetation period. The intensity of colonization and the development of arbuscules were maintained at low values, with a peak of arbuscules at cob formation (3.67) for the control variant and at the 6-leaf phenophase for the treated variant. The correlation between the mycorrhization parameters indicates a very close interconnection between the colonization strategy and the developed structures, with a maximum correlation of 0.91 between frequency and intensity. Mycorrhizal patterns show strong variations between variants and phenophases. Untreated plants showed a longitudinal colonization with a lateral development of arbuscules in the 6-leaf and 8–10-leaf phenophases, compared to fertilized plants, where the colonization was oriented toward point development of both hyphae and arbuscules in the same phenophases. Overall, the application of fertilizers produces a shift in the colonization strategy, from longitudinal to point-irregular in the case of the control variant, and to constant radial colonization in the fertilized variant.

## 5. Materials and Methods

### 5.1. Field Location and Experimental Design

The experiment was located in Cojocna, at the Experimental Didactic Resort, Cojocna Farm, of the University of Agricultural Sciences and Veterinary Medicine, Cluj-Napoca. It was located southeast of the city of Cluj-Napoca, (46°44′56″ lat. N and 23°50′0″ long. E), in Cluj County, Romania. The biological material studied was the MAS 24.C (http://www.maisadour-semences.fr/, accessed on 10 November 2021) corn hybrid. This hybrid is suitable for all soil types, being very tolerant of environmental factors. It belongs to the FAO 270 group. The size of the plot was 100 square meters with 3 replications for each variant. The climate of the experimental area was continental temperate, the annual average temperature being +9.6 °C, with a minimum in January of −5 °C, and a maximum in July/August of +20 °C. The maximum temperature observed can reaches values exceeding 34–35 °C, and the absolute minimum is −35 °C. The average annual rainfall is 866 mm. The determined soil type is phaeoziom, with a loamy texture. 

Soil basic properties are: pH: 6.61; Ca^2+^ me/100 g soil: 9.4; Mg^2+^ me/100 g soil: 1.4; K ppm: 345; P ppm: 53.4; N-NO3 ppm: 3.60; N-NH4 ppm: 22.80; N-min ppm: 26.40.

A bifactorial experiment was placed in the spring of 2020. The entire experiment analyzed changes in mycorrhizal colonization due to fertilization along a developmental gradient. The control point of the experiment represented the colonization at the moment of fertilizer application (A0) in the 2–4-leaf stage (B1). This led to a combination coded in the text as A0–B1. Factor A represents fertilization, with 2 gradations: A1—unfertilized and A2—fertilized with the biostimulator-product AMER 6.3 (1 L ha^−1^). The treatment was used to identify potential changes in root mycorrhizal colonization at the level of strategy or development mechanism. Due to its high content of amino acids, AMER 6.3 positively influences the growth of plants and increases plants’ tolerance to stress caused by various environmental factors. It stimulates the development of roots, which have much greater access to the absorption and assimilation of nutrients. Furthermore, due its high concentration of amino acids, at 39.4%, this biostimulator also helps to synthesize chlorophyll (https://www.agrimedia.ro/, accessed on 9 November 2021). 

Factor A includes fertilization with two gradations: the unfertilized variant A1 and the variant A2 fertilized with the biostimulator-product AMER 6.3. (A0, mycorrhization at point 0 and time of application of biofertilizer, 2–4-leaf phenophases). Factor B represents agronomic indicators regarding the period of plant development (phenophase) and includes 5 gradations: B1—2–4 leaves (as a control point for the start of mycorrhizal colonization); B2—6 leaves; B3—8–10 leaves; B4—cob formation; B5—physiological maturity. Root colonization was studied in all 5 phenophases of the plant. The code A1 and A2 were applied only for stages B2–B5, in order to assess the changes in colonization dynamics and the potential divergence in this mechanism. Roots samples were collected from 5 plants at each phenophase. All roots from each variant were washed in the field prior to cleaning and staining procedures.

### 5.2. Laboratory and Microscopic Analysis

After harvesting the maize roots, they were brought to the microbiology laboratory and prepared for the determination of mycorrhizal colonization parameters. For the microscopic analysis of maize roots, the staining method described by [47] was used, in which agents such as ink, vinegar, and caustic soda are used to observe the intraradicular structures of mycorrhizal fungi. Modifications to this method have been made by [48], which include 4 steps, namely:

1. Cleaning step: this consists of immersing the roots in 10% NaOH solution for 24–48 h; the time varies depending on the species, but also on the development of the root;

2. Rinse step: after letting them stand in the 10% NaOH solution, rinse the roots several times with distilled water;

3. Staining step: this involves immersing the roots for 24 h in a solution of 5% ink (Pelikan blue) and 5% white vinegar (which comes from food vinegar with 9% acetic acid). Discoloration step: After 24 h, the roots were rinsed for 10–20 min with distilled water or for 3 min with pure vinegar.

4. At the end of the stages, the roots were prepared for microscopic analysis. Pieces of root 1 cm in size were used. The MycoPatt methodology was used, which ensures the objective evaluation of the mycorrhizal level in the root cortex by allowing the change of the observation angle by using the grids from the microscopic eyepiece (10 × 10) or the microscopic image analysis system.

Thus, colonization maps can be created at different levels of resolution that report to the exact root length. MycoPatt evaluates in detail how mycorrhizae are extended and each indicator is calculated separately horizontally and vertically for each microscopic field. In this way, the final data report was made objectively and shows the real extent of the structures in the roots [36,49].

### 5.3. Data Analysis

The entire data base consisted of 5850 observations and all data analyses were conducted with different packages available in R Studio software version, 1.4.1106 [50], under the R platform [51]. All histograms were developed within the package “graphics” [51] in order to identify where are the most values were positioned and to observe their extension. Basic statistics were extracted with the package “psych” [52], with further use of means and standard errors (s.e.) for variance and multiple comparison analysis. There were 450 observations used for the control point A0–B1 and 675 observations for each of the rest of the factor combinations. The interactions between parameters were analyzed with scatterplots, and regressions were calculated for each bi-factorial interaction with the “stats” package from the R platform [51]. Regressions were further verified with the “MASS” package [53,54]. Pearson correlations between parameters were calculated with the package “Hmisc” and all significant correlations were analyzed [55]. Differences between parameters, grouped by treatments and phenopases, were analyzed with ANOVA and LSD tests within the package “agricolae” [56]. The entire set of mycorrhizal parameters was compared with principal component analysis (PCA) available in the package “vegan”, with the best solution presenting the highest sum of variance within the first two axes [57] All PCA solutions were verified with non-metric multidimensional scaling (NMDS) available in the same package.

## Figures and Tables

**Table 1:** Exploration of treatment and phenophase interaction on mycorrhizal colonization.

	Frequency (%)	Intensity (%)	Arbuscules (%)	Vesicles (%)	Colonization Degree (%)	Non-Mycorrhizal Areas (%)	Mycorrhizal/Non-Mycorrhizal Report
A0–B1	13.34 ± 0.98 ^g^	6.08 ± 0.44 ^e^	1.04 ± 0.22 ^de^	0.16 ± 0.05 ^ab^	2.63 ± 0.38 ^g^	93.92 ± 0.44 ^a^	0.08 ± 0.01 ^e^
A1–B2	19.65 ± 0.94 ^f^	9.05 ± 0.43 ^d^	1.79 ± 0.19 ^cd^	0.01 ± 0 ^c^	4.31 ± 0.39 ^fg^	90.95 ± 0.43 ^b^	0.13 ± 0.01 ^de^
A1–B3	29.82 ± 1.23 ^e^	13.5 ± 0.6 ^c^	2.93 ± 0.27 ^ab^	0.28 ± 0.05 ^a^	8.6 ± 0.59 ^cd^	86.49 ± 0.6 ^c^	0.22 ± 0.02 ^abc^
A1–B4	44.57 ± 1.17 ^b^	19.23 ± 0.58 ^a^	3.67 ± 0.26 ^a^	0.02 ± 0.01 ^c^	12.59 ± 0.6 ^ab^	80.77 ± 0.58 ^e^	0.31 ± 0.02 ^a^
A1–B5	50.17 ± 1.1 ^a^	20.25 ± 0.49 ^a^	1.02 ± 0.13 ^de^	0.08 ± 0.02 ^bc^	13.3 ± 0.53 ^a^	79.74 ± 0.49 ^e^	0.3 ± 0.01 ^a^
A2–B2	22.98 ± 1.11 ^f^	10.72 ± 0.53 ^d^	3.37 ± 0.34 ^ab^	0.11 ± 0.03 ^bc^	6.16 ± 0.52 ^ef^	89.28 ± 0.53 ^b^	0.17 ± 0.01 ^cde^
A2–B3	37.94 ± 1.16 ^c^	16.72 ± 0.58 ^b^	2.55 ± 0.24 ^bc^	0.11 ± 0.03 ^bc^	10.47 ± 0.61 ^bc^	83.27 ± 0.59 ^d^	0.27 ± 0.02 ^ab^
A2–B4	32.56 ± 1.14 ^de^	15 ± 0.55 ^bc^	2.43 ± 0.2 ^bc^	0.1 ± 0.03 ^bc^	8.72 ± 0.55 ^cd^	85 ± 0.55 ^cd^	0.29 ± 0.05 ^a^
A2–B5	37.33 ± 1.04 ^cd^	14.8 ± 0.38 ^bc^	0.09 ± 0.04 ^e^	0.06 ± 0.02 ^bc^	7.89 ± 0.39 ^de^	85.2 ± 0.38 ^cd^	0.19 ± 0.01 ^bcd^
FactA	123.03	96.34	9.88	2.12	54.57	96.31	15.86
	*p < 0.001*	*p < 0.001*	*p < 0.001*	*0.120*	*p < 0.001*	*p < 0.001*	*p < 0.001*
FactB	153.82	93.51	51.74	12.26	49.80	93.46	17.85
	*p < 0.001*	*p < 0.001*	*p < 0.001*	*p < 0.001*	*p < 0.001*	*p < 0.001*	*p < 0.001*
FactA:FactB	47.50	34.80	16.16	10.73	27.32	34.77	5.15
	*p < 0.001*	*p < 0.001*	*p < 0.001*	*p < 0.001*	*p < 0.001*	*p < 0.001*	*0.001*

Note: Means ± s.e. followed by different letters present significant differences at *p* < 0.05 according to LSD test. A0–B1 represents the phenophase of 2–4 leaves (as a control point for the start of mycorrhizal colonization). The application of fertilizer leads to four different combinations for each variant (A1—control (unfertilized variant) and A2—treated variant): B2—6 leaves; B3—8–10 leaves; B4—cob formation; B5—physiological maturity.

**Table 2:** Pearson correlations showing the interdependence of mycorrhizal colonization parameters.

	Frequency	Intensity	Arbuscules	Vesicles	Non-Mycorrhizal Areas	Mycorrhizal/Non-Mycorrhizal Report	Colonization Degree
Frequency		0.91	0.54	0.16	−0.91	0.88	0.47
Intensity	0.91		0.68	0.19	−1.00	0.97	0.62
Arbuscules	0.54	0.68		0.18	−0.68	0.69	0.46
Vesicles	0.16	0.19	0.18		−0.19	0.19	0.25
Non-Mycorrhizal Areas	−0.91	−1.00	−0.68	−0.19		−0.97	−0.62
Mycorrhizal/Non-Mycorrhizal Report	0.88	0.97	0.69	0.19	−0.97		0.64
Colonization Degree	0.47	0.62	0.46	0.25	−0.62	0.64	

**Table 3:** Forecast of potential intensity based on observed frequency in different treatments and phenological stages of corn.

Variant	Regression Equation
A0–B1	y = 0.46 + 0.42 × x
All samples	y = 0.59 + 0.42 × x
A1–B2	y = 0.67 + 0.43 × x
A1–B3	y = 0.20 + 0.45 × x
A1–B4	y = −0.24 + 0.44 × x
A1–B5	y = 1.11 + 0.38 × x
A2–B2	y = 0.46 + 0.45 × x
A2–B3	y = −0.56 + 0.46 × x
A2–B4	y = 0.82 + 0.44 × x
A2–B5	y = 2.78 + 0.32 × x

Note: A0–B1 represents the phenophase of 2–4 leaves (as a control point for the start of mycorrhizal colonization). The application of fertilizer leads to four different combinations for each variant (A1—control (unfertilized variant) and A2—treated variant): B2—6 leaves; B3—8–10 leaves; B4—cob formation; B5—physiological maturity.

## Data Availability

The datasets generated during and/or analyzed during the current study are available on reasonable request from the corresponding authors.

## Supplementary Materials

The following are available online at https://www.mdpi.com/article/10.3390/plants10122760/s1, Figure S1: histograms of data distribution and normality in colonization parameters—control vs. treated variant (color code: control—green, treatment—blue): (a,b) frequency (%); (c,d) intensity (%); (e,f) arbuscules (%); (g,h) vesicles (%); (i,j) non-mycorrhizal areas (%); (k,l) colonization degree (%); (m,n) mycorrhizal/non-mycorrhizal report.
