# Supplementary material for: Divergence in Corn Mycorrhizal Colonization Patterns Due to Organic Treatment
Source: Plants (Basel). 2021 Dec 14;10(12):2760. doi: 10.3390/plants10122760 (PMC8708519; doi:10.3390/plants10122760)
Supplement: Supplementary file 1 [file plants-10-02760-s001.zip › plants-1489039-supplementary.pdf]

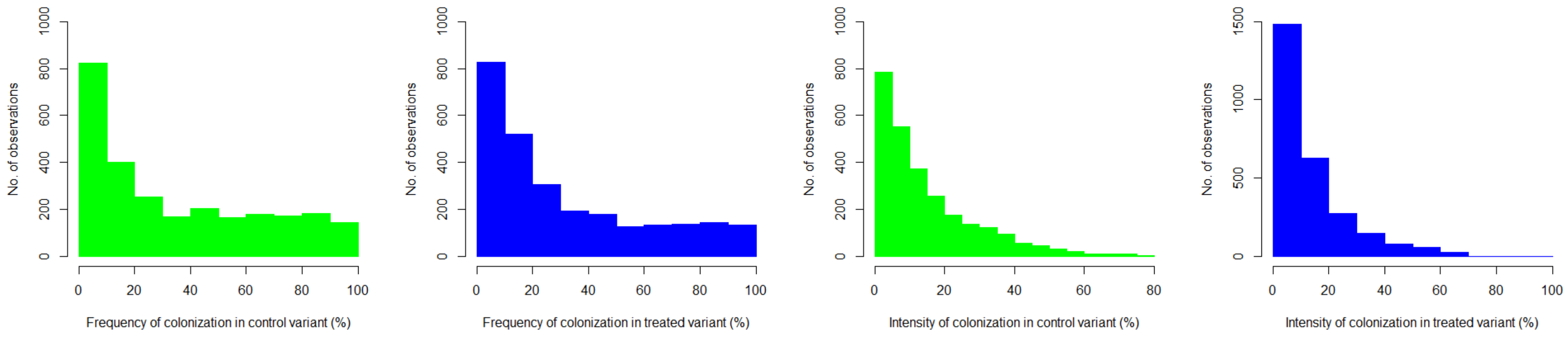

a–b) Frequency

c–d) Intensity

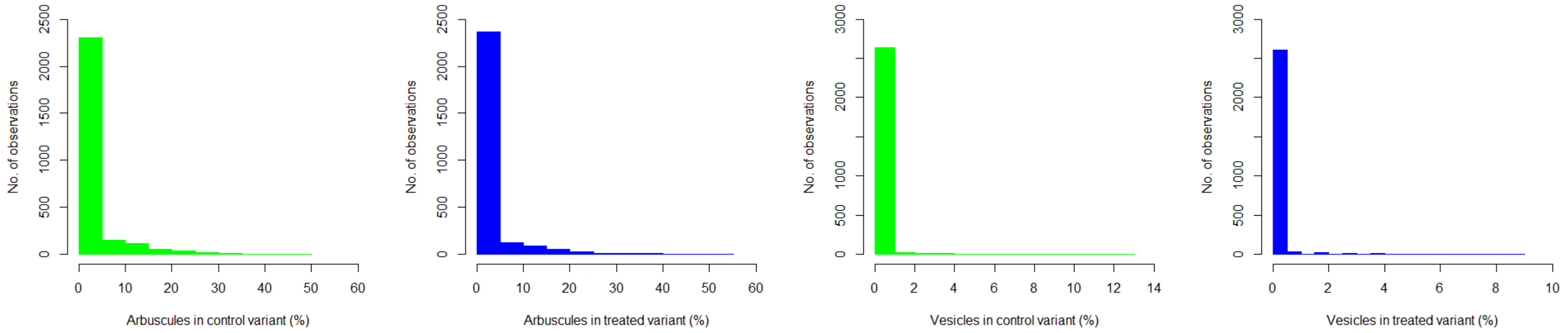

e–f) Arbuscules

g–h) Vesicles

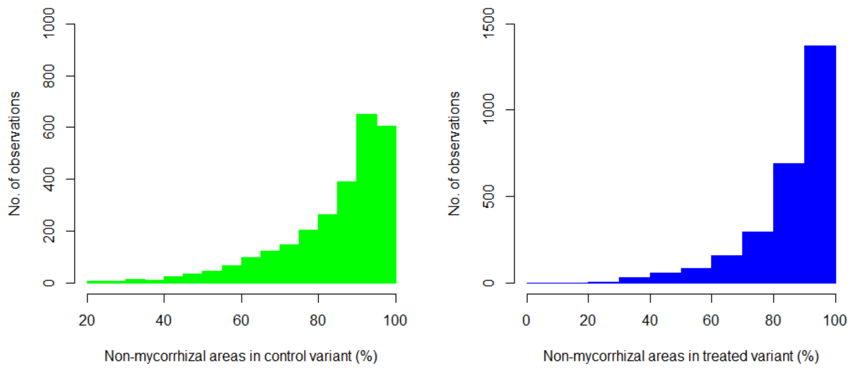

i–j) Non-mycorrhizal areas

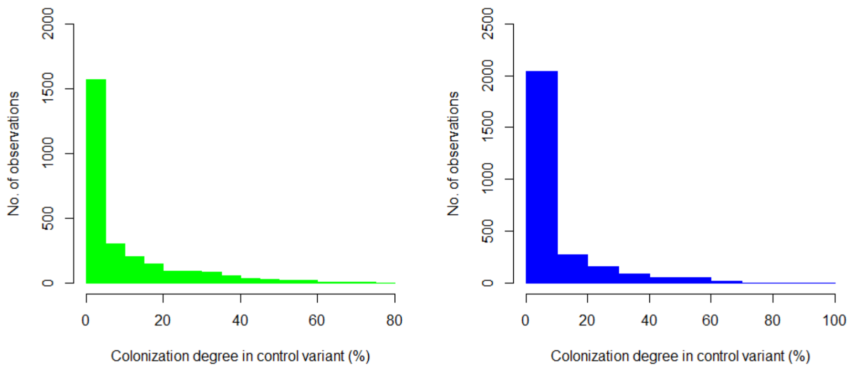

k–l) Colonization degree

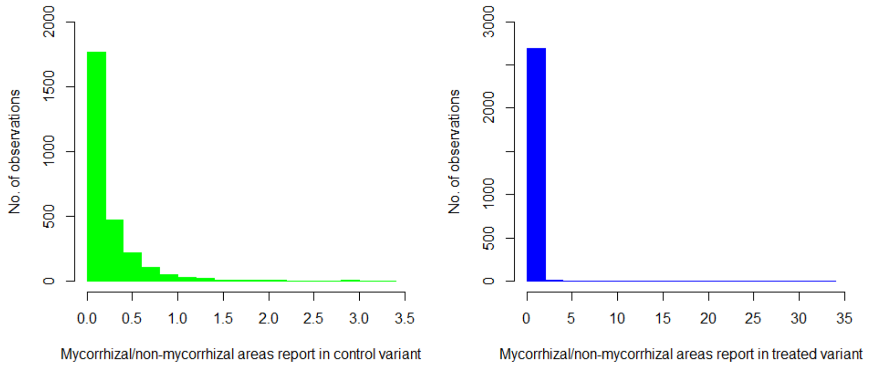

m–n) Mycorrhizal / non-mycorrhizal report

Figure S1. Histograms of data distribution and normality in colonization parameters - Control vs. Treated variant (Color code: Control – green, Treatment – blue): a–b) Frequency (%); c–d) Intensity (%); e–f) Arbuscules (%); g–h) Vesicles (%); i–j) Non-mycorrhizal areas (%); k–l) Colonization degree (%); m–n) Mycorrhizal / non-mycorrhizal report.
